# Vaccine vectors based on Adenovirus 19a/64 exhibit broad cellular tropism and potently restimulate HCMV-specific T cell responses *ex vivo*

**DOI:** 10.1038/s41598-018-19874-1

**Published:** 2018-01-24

**Authors:** Richard Kiener, Markus Fleischmann, Christiane Schwegler, Zsolt Ruzsics, Christian Thirion, Silke Schrödel, Benedikt Asbach, Ralf Wagner

**Affiliations:** 1 Institute of Medical Microbiology and Hygiene, Universität Regensburg, Franz-Josef-Strauß-Allee 11, 93053 Regensburg, Germany; 2 SIRION Biotech GmbH, Am Klopferspitz 19, 82152 Martinsried, Germany; 3 Institute of Virology, Medical Center – University of Freiburg, Medical Faculty, University of Freiburg, Hermann-Herder Str 11, 79104 Freiburg, Germany; 4 Institute of Clinical Microbiology and Hygiene, University Hospital Regensburg, Franz-Josef- Strauß-Allee 11, 93053 Regensburg, Germany

## Abstract

Human Cytomegalovirus (HCMV) remains a major health burden and the development of a vaccine is a global priority. We developed new viral vectors delivering the T cell immunogens IE-1 and pp65 based on Adenovirus 19a/64 (Ad19a/64), a member of subgroup D. In this *ex vivo* study, the novel vectors were compared side by side to Ad5 or modified Vaccinia Ankara (MVA) strains expressing the same transgenes. We found that unlike Ad5, Ad19a/64 vectors readily transduce a broad panel of immune cells, including monocytes, T cells, NK cells and monocyte-derived dendritic cells (moDCs). Both Ad19a/64- and MVA-transduced moDCs efficiently restimulated IE-1 or pp65-specific T cells but MVA induced a higher amount of cytotoxicity in this cell type. Ad5 and Ad19 induced upregulation of CD86 and HLA-DR in moDCs whereas expression of CD80 and CD83 was largely unaltered. By contrast, MVA transduction led to downregulation of all markers. Taken together, our data demonstrate that Ad19a/64 is a promising vector for the delivery of HCMV immunogens since it transduces dendritic cells with an efficiency that is comparable to MVA, but cytotoxicity and interference with dendritic cell maturation are less pronounced.

## Introduction

Human adenoviruses (AdVs) comprise a large family (>70 serotype) of non-enveloped, double-stranded DNA viruses that are subdivided into seven species termed A-G^1–3^. Depending on the serotype, AdV infection can affect the respiratory, gastrointestinal or urinary tract as well as the eye, occasionally causing severe disease. Nonetheless, natural infection with these ubiquitous viruses is mostly asymptomatic or merely accompanied by mild symptoms^4^. Recombinant, replication-defective adenoviruses are extensively utilized as vectors for vaccination, cancer treatment or the delivery of therapeutic genes. Reasons for the popularity of AdV as vaccine vectors include high packing capacity and immunogenicity, combined with an excellent safety profile and the capability to infect both dividing and non-dividing cells^5–8^. Simple and inexpensive methods for vector construction and purification of high titer viral stocks from cell culture further contribute to making the AdV vector platform versatile in use.

Historically, most studies on basic aspects of Adenovirus biology were carried out using AdV type 5 (Ad5, a member of subgroup C), and as a consequence, recombinant vectors were almost exclusively based on Ad5 for many years^9^. However, broad usage of these vectors is limited by preexisting immunity to Ad5 in humans with the presence of neutralizing antibodies (NAbs) reaching up to 90% in some regions^10^. Efficient transduction by Ad5 is also confined to cells expressing the Coxsackie virus and Adenovirus receptor (CAR)^11^. Direct binding to erythrocytes, liver sequestration of virions and hepatotoxicity after intravenous administration constitute additional disadvantages of Ad5-based vectors counteracting broad clinical application^12–14^.

In order to exploit the natural diversity of Adenoviruses and to overcome the limitations of Ad5-based vectors, an increasing number of AdVs from different subgroups has been vectorized in recent years^15^. Vector alternatives like Ad6 (NAb frequency ~68%^10^), Ad26 (NAb frequency ~43–68%^16^) or Ad35 (NAb frequency ~5–18%^16^) were demonstrated to be immunogenic and well tolerated in animal models and humans^17–20^. Beyond that, chimpanzee Adenoviruses (chAdVs) like chAd3 and chAd63 are also emerging as a new vector class, although preexisting immunity in humans (up to 33% NAb frequency for chAd63^21^) has been reported as well^22–24^. While the aforementioned AdV-based vaccine candidates have mostly shown promise in clinical trials, it has also become evident that repeated administration of the same vector is hampered by the induction of neutralizing antibodies^25^. This underlines that novel AdV vectors should still be established to meet an increasing demand for safe and efficacious delivery systems in gene therapy and vaccination^26^.

Previously, an E1/E3-deleted gene therapy vector based on Adenovirus 19a (recently renamed to Ad64^27^, NAb frequency ~16–19%^28,29^), a member of subgroup D that causes epidemic keratoconjunctivitis in humans, has been described^30,31^. AdVs from this subgroup display a particularly broad host cell tropism since they bind to ubiquitously expressed sialic acids rather than CAR^32,33^. In the present study, we wanted to further explore the characteristics of this vector platform by assessing the potential of Ad19a/64 to deliver immunogens from human cytomegalovirus (HCMV).

HCMV is a ubiquitous beta-herpesvirus that represents the most common congenital infection and a major source of complications in transplant recipients^34^. Since HCMV establishes life-long latency and T cell mediated immunity plays a key role in controlling viral replication *in vivo*^35^, there is a demand for the development of therapeutic vaccine candidates that aim at expanding HCMV-specific T cell responses. Such T cell vaccines might contribute to controlling viral latency, thereby reducing the risk of HCMV reactivation in immunocompromised individuals. As novel HCMV T cell vaccine candidates, we generated Ad19a/64 vectors containing IE-1 or pp65, the major targets of T cell mediated immune responses during natural HCMV infection^36,37^. Such vectors could be applied as a therapeutic T cell vaccine or as part of a prophylactic vaccine concept in combination with B cell immunogens such as the fusion protein gB and the pentameric complex.

Here, we compared the Ad19a/64 based vector with its Ad5-based counterpart as well as IE-1- or pp65-containing vectors that are based on the poxvirus strain modified Vaccinia Ankara (MVA), which is currently undergoing clinical testing as a therapeutic HCMV vaccine^38^ in a series of *ex vivo* assays. We were able to confirm the broad tropism of Ad19a/64 by successfully transducing various leukocyte populations. Further, we focused on the impact that Ad19a/64 transduction specifically had on dendritic cells (DCs), because they are the main initiators of adaptive T cell immunity *in vivo*. Moreover, DCs that are manipulated *ex vivo* to present HCMV antigens could be readily applied as a therapeutic vaccine. We found that Ad19a/64 and MVA were both superior to Ad5 in transducing monocyte-derived dendritic cells (moDCs) and mediating antigen presentation. At the same time, induction of apoptosis as well as downregulation of costimulatory molecules from the cell surface was less pronounced for Ad19a/64 compared to MVA. Our data demonstrated that Ad19a/64 might be a promising new HCMV vaccine candidate and a useful vector for gene delivery to human dendritic cells in general.

## Results

**Figure 1:**
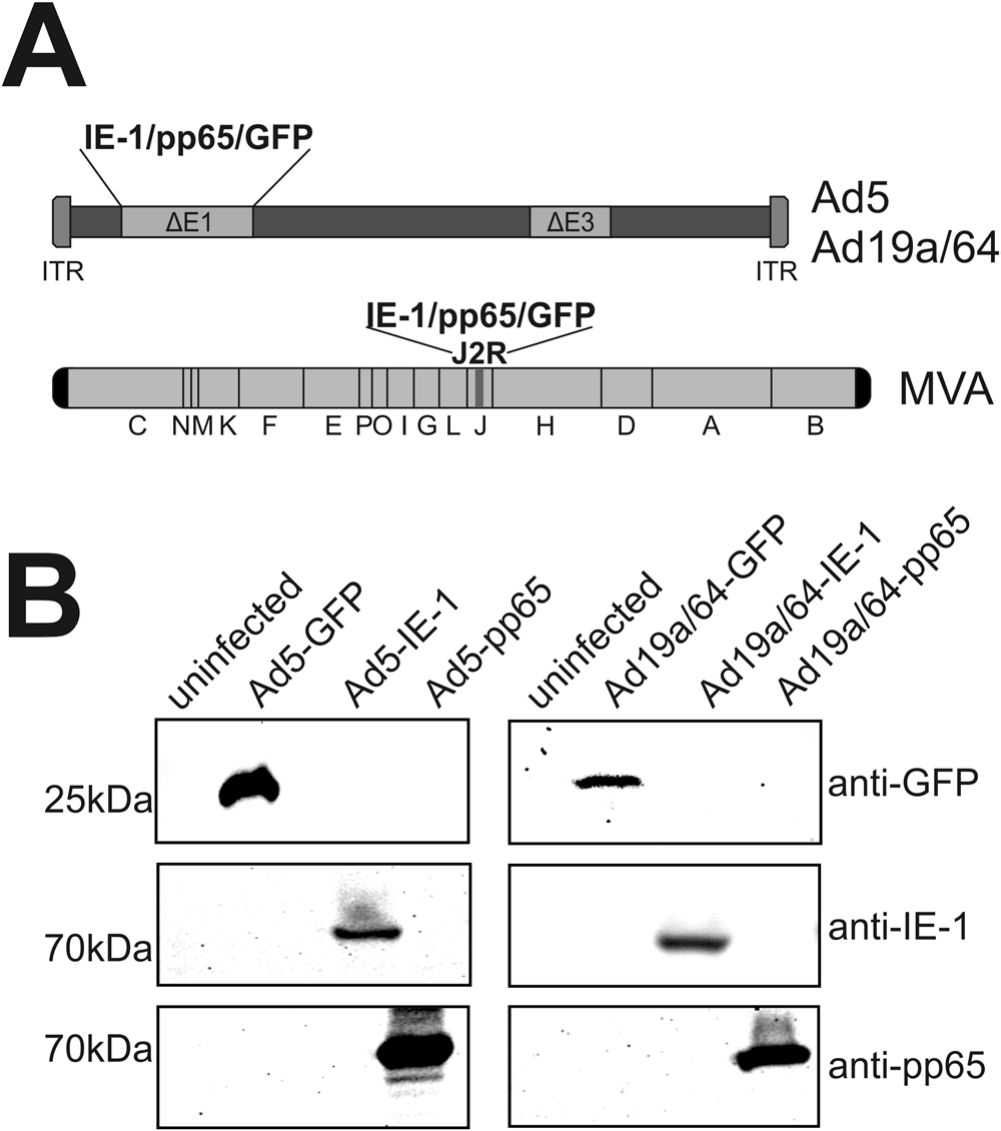
Ad5 and Ad19a/64 vectors mediate transgene expression in permissive cells. (**A**) Schematic representation of AdV and MVA genomes indicating transgene insertion sites. (**B**) Western Blot analysis of transgene expression 48 hours post infection (hpi). HEK293 T cells were infected at a multiplicity of infection (MOI) of 10 with Ad5 or Ad19a/64 vectors expressing the genes IE-1, pp65, or GFP as indicated. Full-length blots are displayed in Supplementary Figure 1.

One major limitation for application of subgroup C vectors like Ad5 is the requirement for CAR expression on the surface of a given cell subset for efficient transduction. This is a particular obstacle for the direct delivery of genetic material to immune cells, where CAR expression is mostly low or entirely lacking^39–41^. By contrast, the tropism of adenoviruses from subgroup D is much broader since they interact with sialic acids that are found on virtually every cell type. Hence, in order to be capable of delivering IE-1 and pp65 directly to antigen-presenting cells (APCs), we based our HCMV vaccine candidates on the subgroup D adenovirus Ad19a/64. We inserted the transgenes IE-1, pp65 or GFP into an E1/E3-deleted vector^31^ at the position of the viral E1-region under the control of the CMV immediate/early promoter (Fig. 1A)^42^. For comparison, the same ORFs were likewise inserted into the E1 region of a BAC derived Ad5 vector under the identical promoter, or the Thymidin kinase locus of MVA under control of a synthetic poxviral early/late promoter. AdV-mediated expression of all transgenes with the expected molecular weight was detectable by Western Blot after infection of permissive HEK293T cells (Fig. 1B).

**Figure 2:**
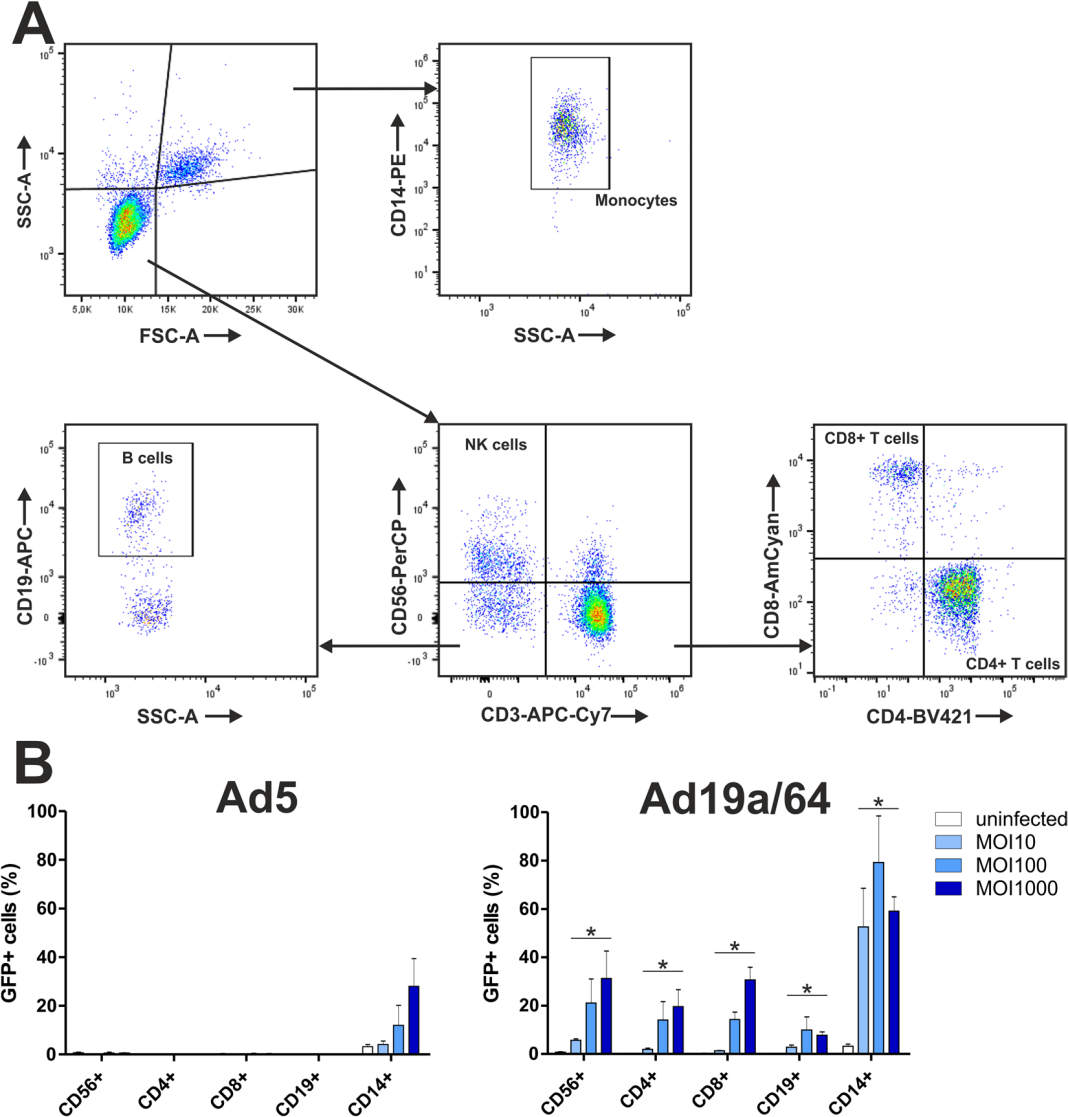
Ad19a/64 vectors are capable of transducing a broad panel of human blood cells. (**A**) Gating strategy to discriminate monocytes (CD14+), B cells (CD19+), NK cells (CD3-/CD56+) and T cells (CD4+ or CD8+). (**B**) Human peripheral blood mononuclear cells (PBMCs) from 3 different donors were transduced at the indicated MOIs with Ad5-GFP or Ad19a/64-GFP. The amount of GFP positive cells in the indicated populations was determined by flow cytometry at 24 hpi. Bars represent the mean and standard deviation (SD) of values from all donors. Asterisks indicate p-values below 0.05 (Bonferroni-corrected Wilcoxon signed rank test) when comparing Ad5 or Ad19a/64 transduced cells in a given subset paired for each donor and MOI.

In a first step, we wanted to investigate the capacity of Ad19a/64 and Ad5 to transduce different leukocyte populations. To identify susceptible cell types, freshly isolated human peripheral blood mononuclear cells (PBMCs) were first infected directly with Ad19a/64-GFP or Ad5-GFP. Then, 24 hours post infection (hpi), the amount of GFP positive cells in various PBMC sub-populations that was defined via staining of the surface markers CD14, CD19, CD3, CD4, CD8 and CD56, followed by flow cytometry analysis (Fig. 2A and B). After transduction with Ad5, GFP expression was only detectable in monocytes, with approximately 30% of cells being positive at a multiplicity of infection (MOI) of 1000. In contrast to this, all populations tested were GFP positive when transduced by Ad19a/64. The highest transduction rates were observed in monocytes (80% positive at MOI 100), followed by NK cells, CD8+ T cells (30% each at MOI 1000) and CD4+ T cells (20% at MOI 1000). Even B cells were transduced with an efficiency of 10% at MOI 100, which however, did not increase upon further increasing the vector load, suggesting that only a fraction of the CD19 positive cell population is susceptible to Ad19a/64 transduction. While there was a considerable variation between the efficacy of transduction within different cell populations, this data confirms that Ad19a/64 is indeed more efficient at delivering genes to leukocytes than Ad5.

**Figure 3:**
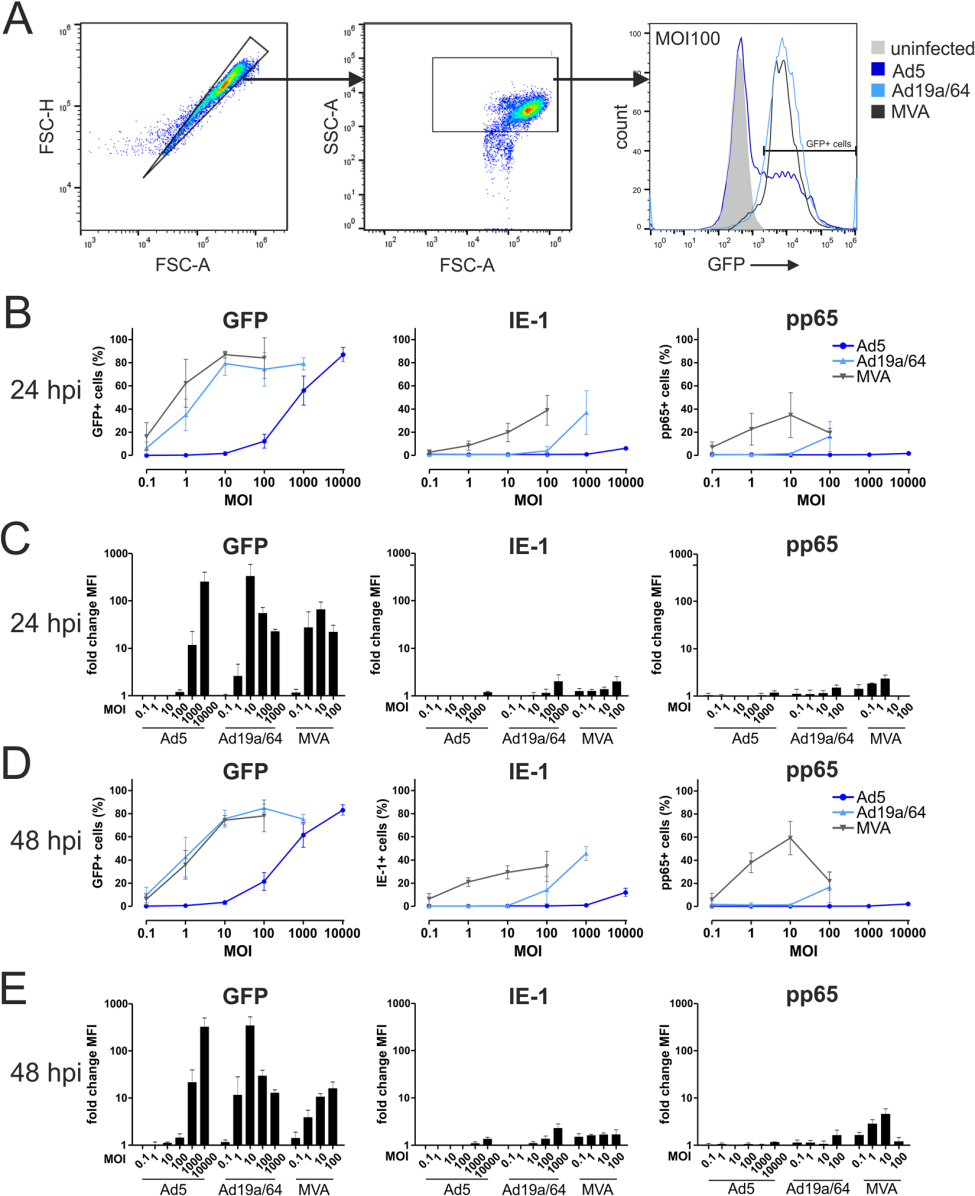
MVA and Ad19a/64 efficiently transduce dendritic cells. MoDCs from 3 different HCMV seronegative blood donors were infected at varying MOIs with the indicated vectors and intracellular presence of the transgenes GFP, IE-1 and pp65 was quantified via flow cytometry after 24 (**B**,**C**) and 48 hours (**D**,**E**). Panel A shows the gating strategy as well as a representative histogram from one donor 48 hours after transduction of moDCs with GFP expressing vectors at MOI 100. Summarized results are given as the median percentage of cells positive for each antigen (**B**,**D**) with error bars representing the standard deviation. Median fluorescence intensity (MFI) values were normalized to the signals obtained from uninfected cells with bars representing the mean and standard deviation of values from all donors (**C**,**E**). Histograms displaying IE-1 and pp65 expression individually for each donor are shown in Supplementary Figure 2.

Since various leukocyte populations were generally found to be susceptible to Ad19a/64 transduction, we next wanted to test whether this vector is capable of delivering HCMV antigens to dendritic cells (DCs). Inducing antigen expression directly in this cell type by use of a suitable vector system might increase the magnitude of adaptive immune responses following vaccination as DCs are the most potent initiators of adaptive immune responses *in vivo*. To assess transduction rates, moDCs were generated *ex vivo* and infected at different MOIs with viral vectors expressing IE-1, pp65 or GFP. Antigen expression was assessed via flow cytometry after 24 and 48 hours, respectively (Fig. 3). Both MVA and Ad19a/64 efficiently transduced moDCs and mediated GFP expression with the maximum (approximately 80% of cells GFP positive) reached at MOI 10. While MVA was slightly superior to Ad19a/64 in mediating GFP expression at low MOIs (MFI at MOI of 0.1 and 1; Fig. 3C and E), the median fluorescence intensity (MFI) values at MOI 10 were higher when Ad19a/64 was used, indicating greater protein levels per transduced cell. Using Ad5, similar transduction rates were observed only at 100–1000-fold higher MOIs of 10,000, demonstrating that, compared to Ad19a/64, this vector is clearly limited in the transduction of moDCs. Due to the limited sensitivity of the monoclonal antibodies used in flow cytometry, the intracellular detection of IE-1 and pp65 was altogether less sensitive compared to GFP, but Ad19a/64 was again clearly superior to Ad5 in mediating antigen expression (Fig. 3, Supplementary Figure 2). At low MOIs, the percentage of antigen positive cells was higher for IE-1 and pp65 when MVA was used compared to Ad19a/64, although the corresponding values were very similar for GFP. Since the MFI values at low MOIs indicate higher protein quantities per cell for MVA, more events are probably capable of surpassing the signal threshold under these conditions, which might explain these discrepancies.

**Figure 4:**
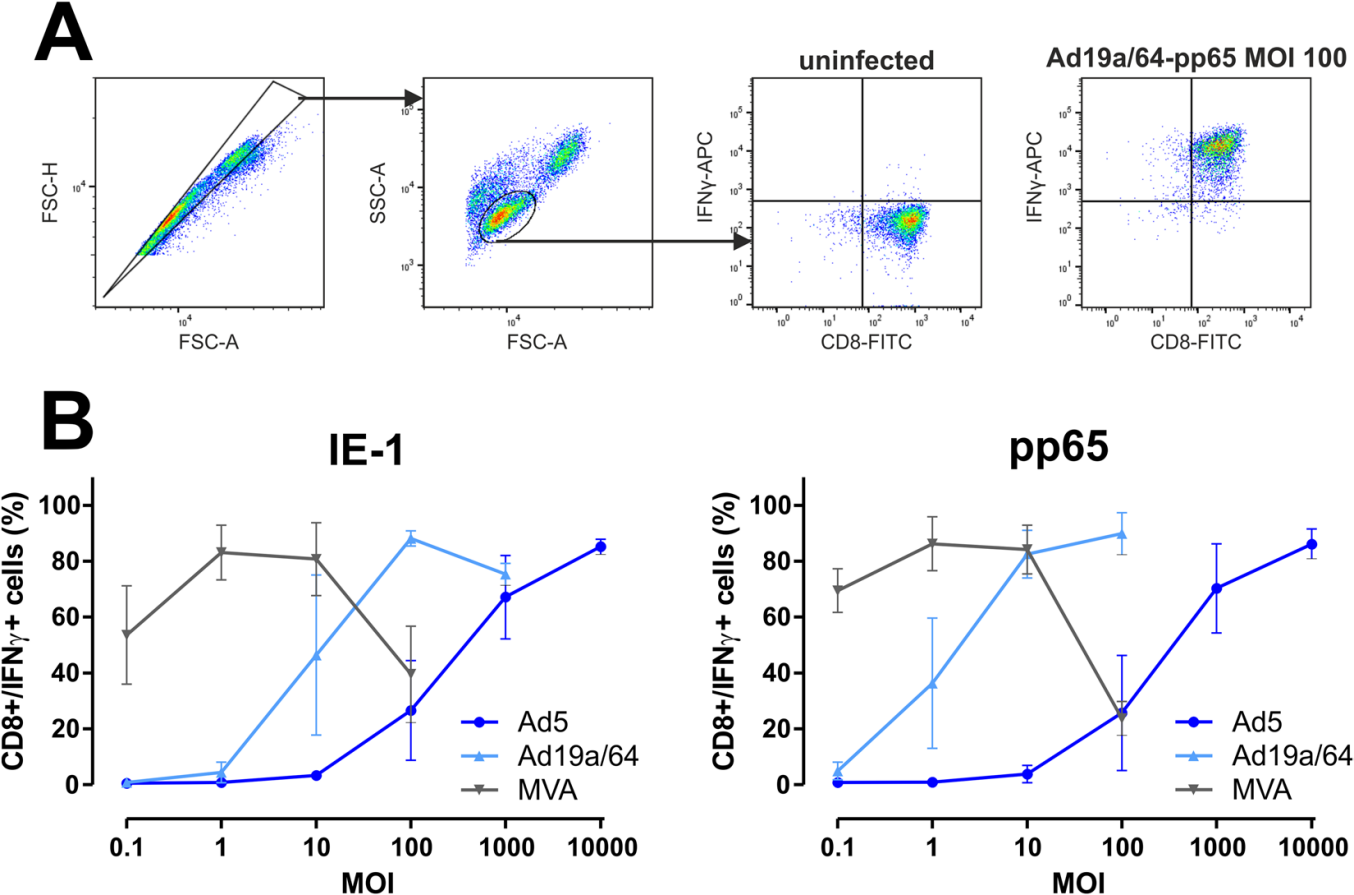
T cell stimulation by virally transduced DCs correlates with transduction rates. MoDCs from 3 different HCMV seronegative blood donors were infected at varying MOIs with the indicated vectors expressing IE-1 or pp65. 24 hpi, antigen-specific, HLA-matched T cell clones were added at an effector/target ratio of 1:1. After 6 hours of co-cultivation in the presence of Brefeldin A (BFA), cells were stained for CD8 and intracellular IFNγ and subjected to flow cytometry analysis. (**A**) Panel a shows the gating strategy employed to identify CD8/IFNγ positive cells. (**B**) The fraction of cells co-expressing CD8 and IFNγ is displayed as the mean and standard deviation of values from all donors.

Recognition of pathogen-derived peptides by T cells requires antigen processing and presentation on major histocompatibility complex (MHC) molecules. Thus, for *in* vivo priming of naïve T cells, MHC presentation of a given peptide is a critical prerequisite. To ensure that there is no major Ad19a/64-mediated interference with MHC-class I presentation, virally transduced moDCs were co-cultivated with CD8 positive, HLA-matched T cell clones recognizing IE-1- or pp65-derived peptides. As an indirect measure for antigen-presentation, T cell restimulation was quantified by intracellular staining of interferon gamma (IFNγ), followed by flow cytometry analysis (Fig. 4). MVA was most efficient at mediating T cell restimulation, inducing a maximum of 80% IFNγ positive T cells at MOI 1 for both antigens, although at MOI 100, T cell responses began to recede. Following Ad19a/64 transduction, a comparable amount of T cell restimulation was observed at MOI 10 (Ad19a/64-pp65) or 100 (Ad19a/64-IE-1), respectively. Using Ad5 vectors, similar levels were reached only at MOI 10,000, thus turning out to be 10 to 100-fold less potent in restimulating pp65 or IE-1 specific T cell clones. Since the T cell restimulation rates were largely reflective of the transduction rates, we conclude that none of the AdV vectors tested here detectably inhibits MHC-class I presentation.

**Figure 5:**
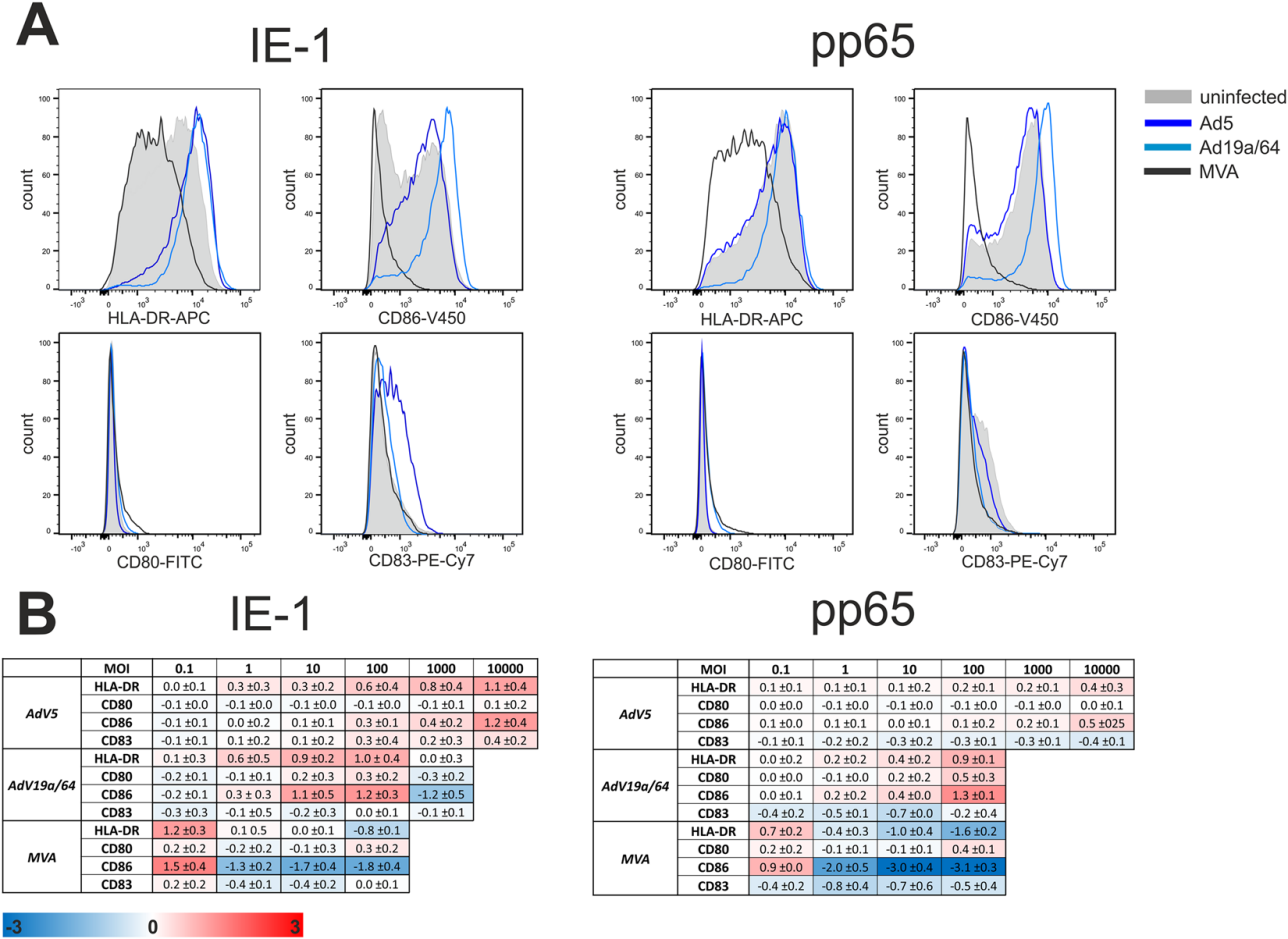
None of the tested vectors mediate full maturation of dendritic cells. MoDCs from 3 different HCMV seronegative donors were infected at various MOIs with IE-1 or pp65-expressing vectors. 48 hpi, samples were stained for HLA-DR, CD80, CD83 or CD86 and analyzed via flow cytometry. Panel A shows representative histograms for one donor after transduction with the indicated vectors at MOI 100. The values from all donors and MOIs are summarized in a heatmap (**B**). Obtained median fluorescence intensity (MFI) values were normalized to those of uninfected cells. Log_2_ values which represent the mean (±standard deviation) from all experiments are displayed in a heatmap indicating up-regulation (red) or down-regulation (blue) of a given marker.

In addition to MHC-presentation of pathogen-derived peptides, priming of naïve T cells requires maturation of dendritic cells, a process during which the surface expression of molecules like CD80, CD83, CD86 and MHC-II is increased. Hence, we wanted to test if any of these markers are upregulated in moDCs by measuring their expression levels 48 hours after transduction (Fig. 5). Transduction with both AdV vectors led to a dose-dependent upregulation of HLA-DR and CD86, except for Ad19 at MOI 1000, where a slight downregulation of CD80 and CD86 was detected. When MVA was used, HLA-DR and CD86 were also upregulated at MOI 0.1, whereas there was a pronounced downregulation of all four markers at higher MOIs with CD86 being affected the most. These data imply that additional inflammatory stimuli will be needed to induce full maturation of dendritic cells, but AdV vectors interfere to a lesser extent with this process than MVA. Additionally, we also measured the secretion of 13 inflammatory cytokines (IL1β, IFNα, IFNγ, TNF, MCP-1, IL-6, IL-8, IL-10, IL-12p70, IL-17, IL-18, IL-23 and IL-33) 48 hours after transduction in a bead-based multiplex assay, but found no significant differences in the secretion profiles elicited by the individual vectors.

**Figure 6:**
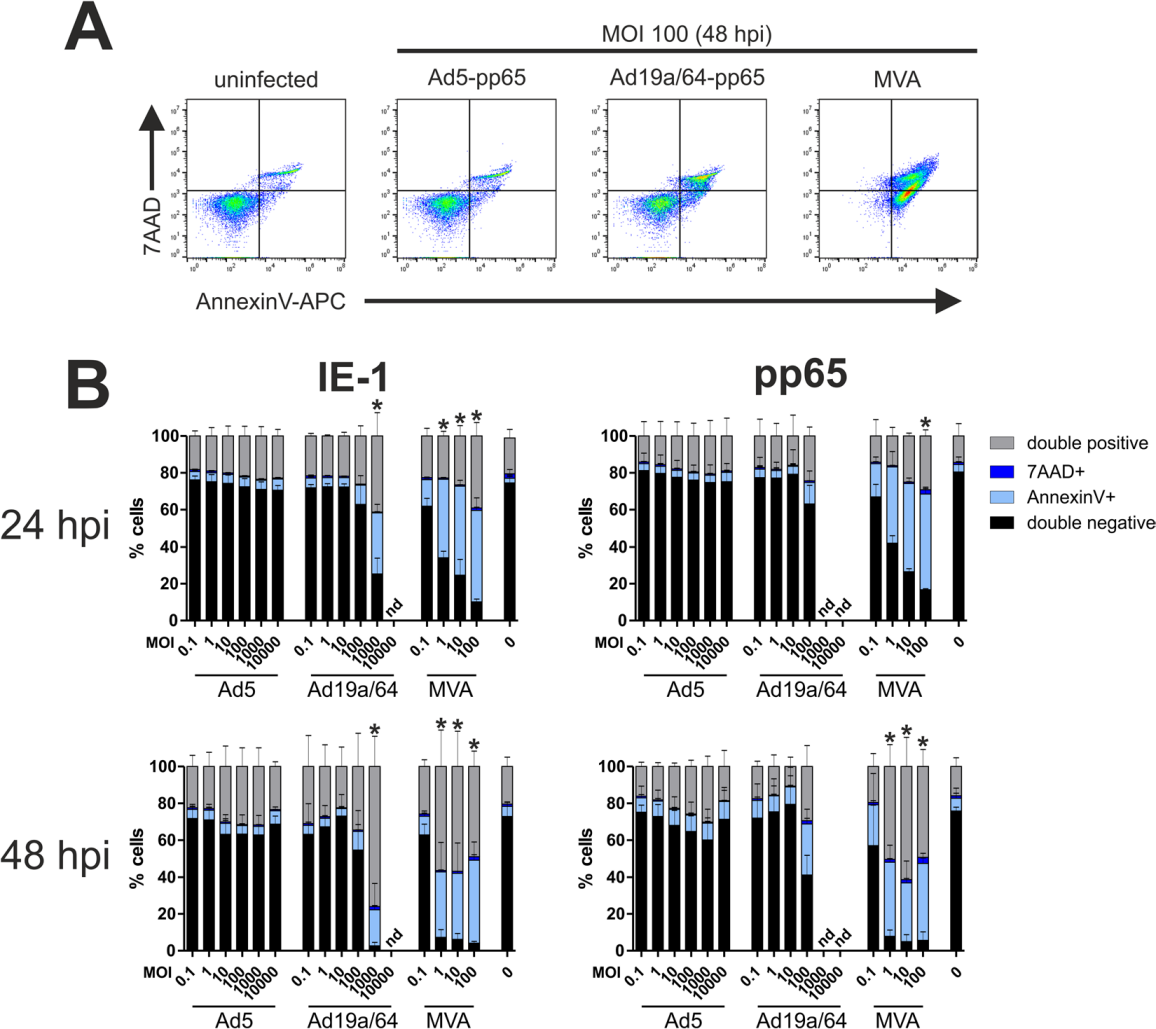
MVA causes the highest amount of cell death in dendritic cells. MoDCs from 3 different HCMV seronegative blood donors were transduced at varying MOIs with the indicated vectors. 24 or 48 hpi, cells were stained with AnnexinV-APC and 7AAD before flow cytometric analysis. Panel A shows representative plots 48 hours after transduction with the indicated vectors at MOI 100. The results from all donors are summarized in (**B**). Bars show the mean and standard deviation (SD) of values from all donors (nd: not determined). Asterisks indicate conditions where the fraction of viable (double negative) cells was significantly (p < 0.05, Bonferroni-corrected t-test) reduced as compared to the uninfected control.

Many viruses induce apoptosis upon infection of dendritic cells, which can in turn impede T cell stimulation when using *ex vivo* transduced DCs as a therapeutic vaccine. Since premature death of virally transduced dendritic cells might limit their therapeutic potential, we wanted to investigate the impact of MVA and AdV transduction on DC survival. We treated moDCs with IE-1- or pp65-expressing vectors and performed AnnexinV/7AAD staining after 24 and 48 hours (Fig. 6). MVA infection induced the highest amount of cytotoxicity, which was already evident at MOI 0.1. At higher virus concentrations, more than 90% of cells were positive for one or both cell death markers after 48 hours. Ad19a/64-mediated toxicity was detectable only starting from MOI 100 with almost all cells undergoing apoptosis at MOI 1000 over a period of 48 hours. Thus, in contrast to MVA, DC transduction and antigen expression take place at MOI 1 and 10 without apparent cytotoxicity when using Ad19a/64. By contrast, Ad5 transduction hardly induced cytotoxic effects at all over the observed time period although MOIs of up to 10,000 were tested. Generally, the inserted transgene had no obvious influence on cell death induction in this assay.

**Figure 7:**
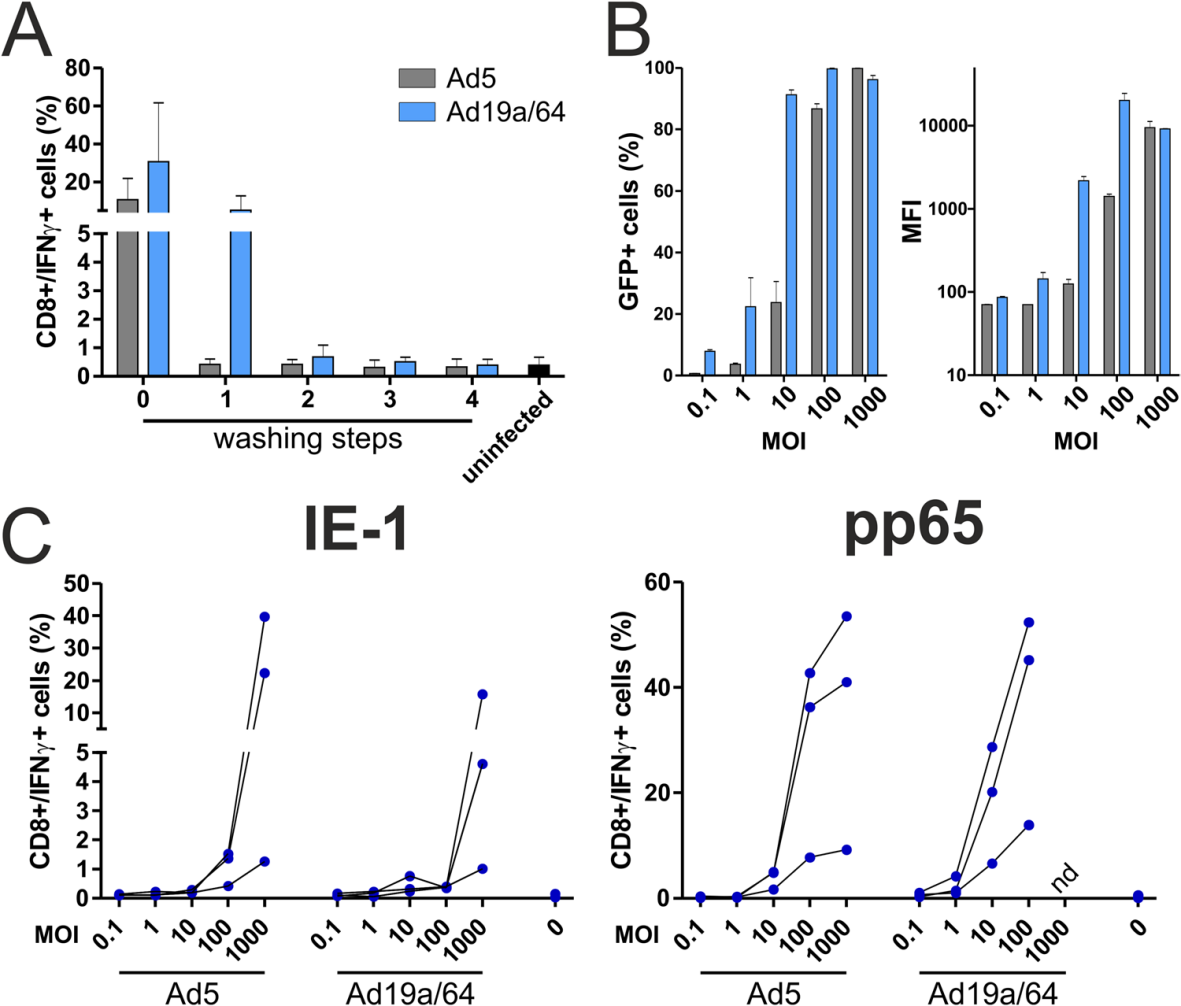
Cross-presentation of IE-1 and pp65 by Ad5 and Ad19a/64-infected cells. (**A**) HeLa cells were transduced at MOI 1000 with Ad5- or Ad19a/64-pp65. 24 hpi, the supernatant from overnight culture (0) as well as from 4 subsequent washing steps with cell culture medium (1–4) was collected and added to moDCs. After 24 hours, a pp65-specific, HLA-matched T cell clone was added to moDCs at an effector/target ratio of 1:1. Co-culture lasted for another 6 hours in the presence of BFA, followed by CD8/IFNγ staining and flow cytometry analysis (the same gating strategy as in Fig. 4 was employed to identify IFNγ positive T cells). Bars represent the mean and SD from 3 experiments with different blood donors. (**B**) HeLa cells were transduced at the indicated MOIs with Ad5-GFP or Ad19a/64-GFP and GFP expression was quantified 24 hpi via flow cytometry. (**C**) HeLa cells were transduced at the indicated MOIs with IE-1 or pp65 expressing AdV vectors. 24 hpi, cells were washed 4 times and added to moDCs at a 1:1 ratio. After 24 hours of co-cultivation, antigen-specific T cell clones were added for a HeLa/DC/T cell ratio of 1:1:1. T cell restimulation was measured after 6 hours as described in A. Connected lines indicate values that were obtained using moDCs from an individual donor (MFI: median fluorescence intensity; nd: not determined).

The majority of currently approved vaccines is administered via intramuscular injection, a scenario in which viral vectors are likely to induce antigen expression predominantly in muscle cells and only to a lower extent directly in tissue-resident dendritic cells. In this scenario, antigen has to be acquired by DCs from the extracellular space and loaded onto MHC-class I molecules via cross-presentation for the priming of CD8+ T cells. To test if AdV-transduced cells or fragments thereof are taken up secondarily by DCs and if MHC-class I presentation takes place, we employed an *in vitro* cross-presentation assay. HeLa cells were transduced with AdV vectors expressing IE-1 or pp65 and 24 hpi, they were added to moDCs. After 24 hours of co-culture, antigen-specific T cell clones were added and the intracellular presence of IFNγ was quantified after 6 hours by flow cytometry. Direct antigen presentation by HeLa cells can be excluded due to an HLA mismatch with the T cell clones used in this experiment. Prior to the addition of HeLa cells to DCs, they were washed multiple times to ensure that no residual extracellular virus particles were left that might infect DCs directly. To test if these washing steps sufficiently removed the inoculum, the supernatant of each washing step was added separately to moDCs. After 24 hours, antigen-specific T cell clones were added and restimulation was assessed as before. Four washing steps were sufficient to reduce T cell activation to background levels, indicating that no detectable amount of free virus remained in the culture (shown representatively for pp65 vectors in Fig. 7A). Using GFP-expressing vectors, we found that HeLa cells are susceptible to infection with both AdV vectors, although Ad19a/64 was again superior to Ad5 in mediating GFP expression (Fig. 7B). Nonetheless, at MOI 1000, almost all HeLa cells were GFP positive irrespective of the vector used with comparable MFI values. Accordingly, the degree of cross-presentation induced by Ad5 or Ad19a/64 transduced cells was generally very similar (Fig. 7C). This finding also implies that many of the differences between Ad5 and Ad19a/64 that were observed in this study are a direct result of their different cell tropism.

## Discussion

It is well established that T cell responses are critical in controlling latent HCMV infection and as a consequence, inducing or expanding T cell responses is a major goal of most vaccine concepts^38,43^. HCMV vaccine candidates delivering T cell immunogens like IE-1 and pp65 might be applied as part of a prophylactic vaccine in combination with B cell immunogens to confine viral replication if sterile immunity cannot be achieved through the induction of antibody responses. At the same time, T cell vaccines can also be used in therapeutic concepts to boost immune responses in patients at risk for HCMV reactivation.

Although Ad5 was repeatedly demonstrated to induce strong T cell responses in various animal models, it has so far proven difficult to overcome preexisting vector immune responses in humans^44^. Results from a prophylactic HIV vaccine phase IIb efficacy trial (STEP trial), where the HIV antigens gag, pol and nef were delivered by Ad5 vectors, even showed increased side effects and higher susceptibility to HIV infection in individuals with high preexisting Ad5 antibody titers^45^. Moreover, subgroup C strains are also inefficient at transducing cells that lack CAR expression. These limitations have spurred the search for alternative AdV vectors in recent years. The subgroup D strain Ad19a/64 that was used as the vector backbone in this study could be a useful alternative to Ad5 due to considerably lower preexisting immunity to this virus in humans^28,29^ and because of its known receptor usage suggesting a broader target cell tropism. Ad19a/64 entry was previously described to be dependent on the presence of common sialic acids as well as the viral knob protein, although the exact binding motif has not been elucidated yet^33^.

We could demonstrate the broad target cell tropism of Ad19a/64 by transducing various leukocyte populations, with the highest transduction rates for monocytes and monocyte-derived dendritic cells^31^. By contrast, transduction of the tested leukocyte subpopulations with Ad5 was either unsuccessful or only achievable at very high MOIs for monocytes and moDCs. This is in accordance with poor or lacking CAR expression on most leukocytes^41^. CAR-independent infection of moDCs by Ad5 has also been reported previously and was suggested to rely on lactoferrin and DC-SIGN^46^. Nevertheless, our data shows that compared to Ad19a/64, such alternative Ad5 entry pathways appear to be orders of magnitude less efficient in mediating the transduction of moDCs.

Compared to Ad5, both Ad19a/64 and MVA were rather efficient at inducing antigen expression in moDCs. While at low MOIs, MFI values were highest for MVA, peak values were higher for the AdV vectors. This might be explained in part by different promoters controlling transcription of GFP, IE-1 and pp65 since in both AdV vectors, the immediate/early promoter of HCMV is used whereas for the MVA vectors, a poxviral synthetic early/late promoter was chosen^42^. At the same time, when using Ad5, much higher MOIs (100–1000-fold) were required for reaching MFI values that were comparable to Ad19a/64.

Consistent with the observed DC transduction rates determined for IE-1- and pp65-expressing vectors, MVA was best at mediating T cell restimulation (particularly at low MOIs), followed by Ad19a/64 and, with some distance, by Ad5. The same trends were observed for both antigens and minor variances between IE-1 and pp65 are likely explained by differences in peptide processing and presentation kinetics or T cell receptor avidities of the clones used.

At MOI 100, the T cell restimulation rates were declining for MVA, a phenomenon that was hardly observed even at higher MOIs for the AdV vectors. This reduction in T cell restimulation is probably a direct result of cytotoxicity, since the poxvirus strain was found to induce the highest amount of cell death (evident already at MOI 0.1). Since Ad19a/64 induced detectable cytotoxic effects only starting from MOI 100, the window in which transduction and antigen presentation take place without the induction of cell death appears to be larger for this vector than for MVA. Interestingly, cell death was not above background levels for the tested MOIs using Ad5, although this is likely not a feature of Ad5 in general, but rather due to inefficient entry into moDCs.

In accordance with previously published data, we also found pronounced downregulation of maturation markers following MVA transduction starting from MOI 1^47^. At the same time, it was surprising that CD86 and HLA-DR were upregulated at MOI 0.1. Previous work by Pascutti *et al*. demonstrated that instead of MVA-infected DCs, uninfected bystander cells are increasing the expression of CD86 and HLA-DR, which could explain why we observed upregulation only at MOI 0.1^48^. The same markers were upregulated as a result of AdV transduction in a dose-dependent manner although the expression of CD80 and CD83 was largely unaltered. Thus, during *ex vivo* transduction of dendritic cells, it might be necessary to add additional activation stimuli such as IFNγ or a TLR agonist to induce full maturation of moDCs and boost their stimulatory capacity.

Finally, we found that both Ad5 and Ad19a/64 are capable of transducing HeLa cells (which do express the CAR receptor^49^) at similar rates, with comparable protein levels being obtained at MOI 1000. Accordingly, cross-presentation by DCs co-cultivated with transduced HeLa cells did not differ significantly between the two AdV vectors. In this experimental setting, the amount of antigen produced in HeLa cells seems to be the major determinant of subsequent T cell restimulation rates.

Taking these results together, we propose that Ad19a/64 could be a useful vector for the *ex vivo* delivery of immunogens to dendritic cells, since transduction, antigen expression and -presentation are superior to Ad5 while at the same time, there is (i) no interference with DC maturation and (ii) cytotoxicity is clearly less pronounced than for MVA. The ability to transduce moDCs without inhibiting maturation or inducing apoptosis is a particularly beneficial vector characteristic, since dendritic cells presenting HCMV antigens have great therapeutic potential for immunosuppressed patients such as transplant recipients. Currently, DCs pulsed with pp65 mRNA are even tested in clinical studies for the treatment of glioblastoma^50^, where expression of HCMV antigens is frequently detectable^51,52^.

For more classical vaccination concepts, where intramuscular immunization is the most frequently used delivery route, muscle cells are presumably the main producers of antigen. Since it was previously demonstrated that in contrast to Ad5, Ad19a/64 is also capable of transducing myocytes with high efficiency, it is likely that secondary antigen uptake by dendritic cells will be higher *in vivo* when this vector is used^33^. At the same time, direct *in vivo* transduction of dendritic cells is also a feasible route for antigen delivery when using Ad19a/64 or MVA.

Our results provide evidence that vectors based on Ad19a/64 are promising tools for the delivery of CMV antigens and that further *in vivo* testing of the described immunogens as vaccine candidates in appropriate animal models is warranted. Beyond that, the favorable vector characteristics of Ad19a/64 could also be exploited for other applications such as prophylaxis or treatment of other chronic infections associated e.g. with various tumor diseases as well as the delivery of tumor antigens for immunotherapy of cancer.

## Methods

### Ethics Statement

Blood donations were collected from healthy, adult volunteers who gave written informed consent beforehand. Sample collections and experiments were approved by the ethics committee of the University of Regensburg and performed in accordance with all relevant guidelines (file reference 16–101).

### Cells and viruses

Baby hamster kidney (BHK-21, ATCC #CCL-10), HEK293T and HeLa cells were grown in DMEM medium, supplemented with 100 U/ml penicillin, 100 μg/ml streptomycin (Pen/Strep) and 10% fetal calf serum (FCS). Peripheral blood mononuclear cells (PBMCs) were purified using Ficoll-Paque (GE Healthcare) according to the manufacturer’s instructions. PBMCs and T cell clones were cultured in RPMI 1640 supplemented with 2 mM L-glutamine, Pen/Strep and FCS. Magnetic activated cell sorting (MACS) with CD14 beads (Miltenyi Biotech) was employed according to the manufacturer’s instructions for the isolation of monocytes from PBMCs. Differentiation to moDCs was induced by cultivation over a period of 5–6 days in RPMI 1640 with FCS, Pen/Strep, 2 mM L-glutamine, 1% non-essential amino acids, 1% MEM vitamins, 1 mM sodium pyruvate, 10 µM β-mercaptoethanol (all from Gibco), 1000 U/ml IL-4 and 1000 U/ml GM-CSF (both from Miltenyi Biotech). After 2 days, the medium was replenished and differentiation was verified via flow cytometry at day 5 or 6 by checking the down-regulation of CD14 and the up-regulation of CD1a. The CD8+ T cell clones 4G6 (specific for the pp65-peptide TPRVTGGGAM on HLA-B7) and 1C3 (specific for the IE-1-peptide QIKVRVDMV on HLA-B8) were a kind gift from Dirk Busch (TU Munich). They were obtained by limiting dilution of PBMCs from CMV seropositive donors, followed by co-culture with feeder cells as described in Nauerth *et al*.^53^. The strain MVA-GFP was kindly provided by Mariano Esteban (CSIC Madrid) and all Ad19a/64 vectors are based on an Ad19a ME strain-derived BAC-cloned vector^31,54^.

### Generation of recombinant MVA vectors

MVA-IE-1 and MVA-pp65 were generated by inserting IE-1 (accession no. AAR31448.1) or pp65 (accession no. P06725.2), which are controlled by a synthetic early/late-promotor^42^, into the TK locus (J2R) of MVA via homologous recombination (described in detail by Perdiguero *et al*.^55^). Briefly, the transgenes were inserted into the vector pLZAW1 containing DNA segments for targeting the TK locus as well as the reporter gene *lacZ*. For homologous recombination, BHK cells were simultaneously transfected with the plasmids and infected with MVA-GFP. Recombinant virus strains were separated from the parental strain MVA-GFP in several rounds of plaque-purification via blue-white screening. Sanger sequencing of the TK locus and Western Blot analysis were performed to ensure correct integration of IE-1 and pp65 as well as protein expression. High-titer virus stocks were generated by large-scale infection of BHK cells, cell lysis by 3 subsequent freeze/thaw cycles and purification of viral particles in two rounds of ultracentrifugation over a 30% sucrose cushion.

### Generation of recombinant AdV vectors

Ad5 and Ad19a/64 vectors were generated as previously described in Ruzsics *et al*.^56^. Briefly, the Gene of Interest (GOI) IE-1, pp65 or eGFP were cloned into the shuttle vectors pO6-A5-HCMV-gfp and pO6-19a-HCMV-MCS, respectively, under the control of an HCMV promoter. The HCMV-GOI-SV40-pA was then transferred via Flp-recombination in *E. coli* into the respective BAC vectors containing the genome of E1/E3 deleted replication deficient Ad5 or Ad19a-based vectors. Recombinant viral DNA was released from the purified BAC-DNA by restriction digest with Pac I. The obtained linear DNA was transfected into 293 cells for virus propagation. Viral vectors were released from cells via NaDeoxycholate extraction. Residual free DNA was digested by DNase I. Afterwards, vectors were purified by CsCl gradient ultracentrifugation followed by a buffer exchange to 10 mM Hepes pH 8.0, 2 mM MgCl_2_ 4% Sucrose via PD10 columns (GE). Titration was performed based on RapidTiter method by detection of infected HEK 293 cells via immunohistochemical staining with anti-hexon antibody (Ad5: Santa Cruz, Ad19a: Novus). Insert integrity was confirmed by PCR amplification of the GOI in DNA purified from the purified vectors. Functionality of the insert was confirmed by qPCR and Western blot of infected NIH3T3 or 293 cells.

### Viral transduction

PBMCs or moDCs were infected by switching the cell culture medium to RPMI 1640 not containing any supplements. Viral stock solutions were likewise diluted in RPMI 1640 and added to the cells. 3 hpi, the medium was removed and infected cells were cultivated in RPMI 1640 with FCS, Pen/Strep and 2 mM L-glutamine until analysis.

### Flow cytometry analysis

Data was acquired using a FACS Canto II device (BD Biosciences) or an Attune NxT acoustic focusing cytometer (Life Technologies). Data was analyzed using FlowJo Version 10. Doublets were first excluded in a FSC-A/FSC-H plot and cells debris was excluded according to FSC/SSC properties.

### Western blot analysis

Cell extracts from HEK293T cells were separated by SDS-PAGE, blotted onto nitrocellulose membranes and proteins were detected with the following antibodies: anti-IE-1 (Abcam, clone IE1.G10), anti-pp65 (Abcam, ab49214) or anti-GFP (SantaCruz, sc-8334) as primary antibodies; anti-mouse IgG-HRP (Jackson Immunoresearch) or anti-rabbit IgG-HRP (Dako) conjugates as secondary antibodies.

### Leukocyte tropism

After 24 hours, PBMCs infected with Ad5-GFP or Ad19a/64-GFP were washed twice with wash buffer (PBS with 1% FCS and 1 mg/ml NaN_3_) and stained for 30 minutes with the following antibodies: anti-CD3-APC-Cy7 (Biolegend, clone SK7), anti-CD4-BV421 (Biolegend, clone RPA-T4), anti-CD8-AmCyan (BD Biosciences, clone SK1), anti-CD14-PE (Biolegend, clone 63D3), anti-CD19-APC (Biolegend, clone HIB19) and anti-CD56-PerCP (Biolegend, clone HCD56, all antibodies were diluted 1:60 in PBS). After staining, cells were washed twice with wash buffer and analyzed on a FACS Canto II device.

### Intracellular antigen staining

24 or 48 hpi, moDCs were washed twice with wash buffer, incubated for 30 minutes with Fix/Perm-Solution (PBS with 4% (w/v) paraformaldehyde and 1% (w/v) Saponin) and washed twice with Perm/Wash-Solution (PBS with 0.1% (w/v) Saponin). Staining was performed with the following antibodies (all diluted 1:50 in PBS): anti-IE-1 (Abcam, clone IE1.G10) or anti-pp65 (Abcam, ab53489), followed by PE goat anti-mouse IgG (Biolegend, poly4053). All antibody incubation periods lasted for 30 minutes and cells were washed twice using Perm/Wash after every step. Samples infected with GFP-vectors were only washed twice using wash buffer before measurement. Data was acquired with a FACS Canto II device.

### Restimulation of T cell clones and intracellular cytokine staining

To match the cognate HLA molecule of the T cell clones used, HLA-B7 positive moDCs were transduced with pp65 carrying vectors while for IE-1 vectors, HLA-B8 positive cells were chosen. 24 hours after transduction, T cells were added to moDCs at a 1:1 ratio together with Brefeldin A (BFA, 1 µg/ml). After 6 hours, samples were washed twice using wash buffer, fixed for 30 minutes with Fix/Perm, washed two times with Perm/Wash and stained for 30 minutes with the following antibodies: anti-CD8α-FITC (BD Biosciences, clone RPA-T8) and anti-IFN-γ-APC (Biolegend, clone 4 S.B3, both antibodies were diluted 1:60 in Perm/Wash). After two more washing steps with Perm/Wash, cells were analyzed using a FACS Canto II cytometer. Based on FSC/SSC-properties, moDCs were excluded from analysis and T cells were identified by gating on CD8+ cells. A gate for IFN-γ positive events was defined based on signals from an uninfected sample.

### Analysis of DC maturation

48 hpi, moDCs were washed twice using wash buffer, treated for 30 minutes with 4% (w/v) PFA in PBS, washed two times and incubated for 30 minutes with the following antibodies: anti-CD80-FITC (BD Biosciences, clone L307.4), anti-CD83-PE-Cy7 (Biolegend, clone HB15e), anti-CD86-V450 (BD Biosciences, clone FUN-1) and anti-HLA-DR-APC (Biolegend, clone L243, all diluted 1:60 in PBS). After two more washing steps, cells were analyzed on a FACS Canto II device.

### AnnexinV/7AAD cytotoxicity assay

24 or 48 hours after infection, moDCs were stained using the APC Annexin V Apoptosis Detection Kit with 7-AAD (Biolegend) according to the manufacturer’s instructions. Flow cytometry analysis was performed using an Attune NxT cytometer.

### Cross presentation assay

24 hours after transduction of HeLa cells with AdV vectors, they were washed 4 times with RPMI1640 medium, followed by detachment from cell culture plates with Trypsin/EDTA solution (Pan Biotech). HeLa cells were added to moDCs at a 1:1 ratio. HeLa cells that were transduced with pp65 containing vectors were added to HLA-B7 positive moDCs whereas HLA-B8 positive moDCs were co-cultivated with HeLa cells that were transduced with Ad5-IE1 or Ad19a/64-IE-1. After 24 hours of co-incubation, T cells were added along with Brefeldin A (BFA, 1 µg/ml) at a moDC/T cell ratio of 1:1. After 6 hours of co-incubation, cells were stained for CD8 and IFNγ and analyzed as described above.

### Data availability

The datasets generated during and/or analyzed during the current study are available from the corresponding author on reasonable request.

## Electronic supplementary material

Supplementary Information

## References

[1] Knipe, D. M. & Howley, P. M. *Fields Virology*. (Lippincott Williams&Wilki, 2013).

[2] Hage E, Gerd Liebert U, Bergs S, Ganzenmueller T, Heim A (2015). Human mastadenovirus type 70: a novel, multiple recombinant species D mastadenovirus isolated from diarrhoeal faeces of a haematopoietic stem cell transplantation recipient. J. Gen. Virol..

[3] Yoshitomi H, Sera N, Gonzalez G, Hanaoka N, Fujimoto T (2017). First isolation of a new type of human adenovirus (genotype 79), species Human mastadenovirus B (B2) from sewage water in Japan. J. Med. Virol..

[4] Wold WSM, Toth K (2013). Adenovirus vectors for gene therapy, vaccination and cancer gene therapy. Curr. Gene Ther..

[5] Liu J (2008). Magnitude and phenotype of cellular immune responses elicited by recombinant adenovirus vectors and heterologous prime-boost regimens in rhesus monkeys. J. Virol..

[6] Hillgenberg M, Schnieders F, Löser P, Strauss M (2001). System for efficient helper-dependent minimal adenovirus construction and rescue. Hum. Gene Ther..

[7] Harvey B-G (2002). Safety of local delivery of low- and intermediate-dose adenovirus gene transfer vectors to individuals with a spectrum of morbid conditions. Hum. Gene Ther..

[8] Zheng C, Baum BJ, Iadarola MJ, O’Connell BC (2000). Genomic integration and gene expression by a modified adenoviral vector. Nat. Biotechnol..

[9] Benihoud K, Yeh P, Perricaudet M (1999). Adenovirus vectors for gene delivery. Curr. Opin. Biotechnol..

[10] Mast TC (2010). International epidemiology of human pre-existing adenovirus (Ad) type-5, type-6, type-26 and type-36 neutralizing antibodies: correlates of high Ad5 titers and implications for potential HIV vaccine trials. Vaccine.

[11] Bergelson JM (1997). Isolation of a common receptor for Coxsackie B viruses and adenoviruses 2 and 5. Science.

[12] Lyons M (2006). Adenovirus type 5 interactions with human blood cells may compromise systemic delivery. Mol. Ther. J. Am. Soc. Gene Ther..

[13] Green NK (2004). Extended plasma circulation time and decreased toxicity of polymer-coated adenovirus. Gene Ther..

[14] Khare R, Chen CY, Weaver EA, Barry MA (2011). Advances and future challenges in adenoviral vector pharmacology and targeting. Curr. Gene Ther..

[15] Alonso-Padilla J (2016). Development of Novel Adenoviral Vectors to Overcome Challenges Observed With HAdV-5-based Constructs. Mol. Ther. J. Am. Soc. Gene Ther..

[16] Barouch DH (2011). International Seroepidemiology of Adenovirus Serotypes 5, 26, 35, and 48 in Pediatric and Adult Populations. Vaccine.

[17] Milligan ID (2016). Safety and Immunogenicity of Novel Adenovirus Type 26- and Modified Vaccinia Ankara-Vectored Ebola Vaccines: A Randomized Clinical Trial. JAMA.

[18] Kelly C (2016). Chronic hepatitis C viral infection subverts vaccine‐induced T‐cell immunity in humans. Hepatol. Baltim. Md.

[19] Nyombayire J (2017). First-in-Human Evaluation of the Safety and Immunogenicity of an Intranasally Administered Replication-Competent Sendai Virus–Vectored HIV Type 1 Gag Vaccine: Induction of Potent T-Cell or Antibody Responses in Prime-Boost Regimens. J. Infect. Dis..

[20] Penaloza-MacMaster P (2013). Alternative serotype adenovirus vaccine vectors elicit memory T cells with enhanced anamnestic capacity compared to Ad5 vectors. J. Virol..

[21] Nébié I (2014). Assessment of Chimpanzee Adenovirus Serotype 63 Neutralizing Antibodies Prior to Evaluation of a Candidate Malaria Vaccine Regimen Based on Viral Vectors. Clin. Vaccine Immunol. CVI.

[22] Hayton E-J (2014). Safety and tolerability of conserved region vaccines vectored by plasmid DNA, simian adenovirus and modified vaccinia virus ankara administered to human immunodeficiency virus type 1-uninfected adults in a randomized, single-blind phase I trial. PloS One.

[23] Ersching J (2010). Neutralizing antibodies to human and simian adenoviruses in humans and New-World monkeys. Virology.

[24] Ledgerwood JE (2017). Chimpanzee Adenovirus Vector Ebola Vaccine. N. Engl. J. Med..

[25] Mastrangeli A (1996). ‘Sero-switch’ adenovirus-mediated *in vivo* gene transfer: circumvention of anti-adenovirus humoral immune defenses against repeat adenovirus vector administration by changing the adenovirus serotype. Hum. Gene Ther..

[26] Delany I, Rappuoli R, De Gregorio E (2014). Vaccines for the 21st century. EMBO Mol. Med..

[27] Zhou X (2012). Analysis of human adenovirus type 19 associated with epidemic keratoconjunctivitis and its reclassification as adenovirus type 64. Invest. Ophthalmol. Vis. Sci..

[28] Vogels R (2003). Replication-deficient human adenovirus type 35 vectors for gene transfer and vaccination: efficient human cell infection and bypass of preexisting adenovirus immunity. J. Virol..

[29] Aoki K, Tagawa Y (2002). A twenty-one year surveillance of adenoviral conjunctivitis in Sapporo, Japan. Int. Ophthalmol. Clin..

[30] Laibson PR (1975). Adenoviral keratoconjunctivitis. Int. Ophthalmol. Clin..

[31] Ruzsics Z (2006). Transposon-assisted cloning and traceless mutagenesis of adenoviruses: Development of a novel vector based on species D. J. Virol..

[32] Arnberg N, Kidd AH, Edlund K, Olfat F, Wadell G (2000). Initial interactions of subgenus D adenoviruses with A549 cellular receptors: sialic acid versus alpha(v) integrins. J. Virol..

[33] Thirion C (2006). Adenovirus vectors based on human adenovirus type 19a have high potential for human muscle-directed gene therapy. Hum. Gene Ther..

[34] Manicklal S, Emery VC, Lazzarotto T, Boppana SB, Gupta RK (2013). The ‘Silent’ Global Burden of Congenital Cytomegalovirus. Clin. Microbiol. Rev..

[35] Einsele H (2002). Infusion of cytomegalovirus (CMV)-specific T cells for the treatment of CMV infection not responding to antiviral chemotherapy. Blood.

[36] Slezak SL (2007). CMV pp65 and IE-1 T cell epitopes recognized by healthy subjects. J. Transl. Med..

[37] Sylwester AW (2005). Broadly targeted human cytomegalovirus-specific CD4+ and CD8+ T cells dominate the memory compartments of exposed subjects. J. Exp. Med..

[38] Schleiss MR (2016). Cytomegalovirus vaccines under clinical development. J. Virus Erad..

[39] Rebel VI (2000). Maturation and lineage-specific expression of the coxsackie and adenovirus receptor in hematopoietic cells. *Stem Cells Dayt*. Ohio.

[40] Philipson L, Pettersson RF (2004). The coxsackie-adenovirus receptor–a new receptor in the immunoglobulin family involved in cell adhesion. Curr. Top. Microbiol. Immunol..

[41] Horvath J, Weber JM (1988). Nonpermissivity of human peripheral blood lymphocytes to adenovirus type 2 infection. J. Virol..

[42] Chakrabarti S, Sisler JR, Moss B (1997). Compact, synthetic, vaccinia virus early/late promoter for protein expression. BioTechniques.

[43] Klenerman P, Oxenius A (2016). T cell responses to cytomegalovirus. Nat. Rev. Immunol..

[44] Zaiss AK, Machado HB, Herschman HR (2009). The influence of innate and pre-existing immunity on adenovirus therapy. J. Cell. Biochem..

[45] Buchbinder SP (2008). Efficacy assessment of a cell-mediated immunity HIV-1 vaccine (the Step Study): a double-blind, randomised, placebo-controlled, test-of-concept trial. Lancet.

[46] Adams WC (2009). Adenovirus serotype 5 infects human dendritic cells via a coxsackievirus-adenovirus receptor-independent receptor pathway mediated by lactoferrin and DC-SIGN. J. Gen. Virol..

[47] Engelmayer J (1999). Vaccinia virus inhibits the maturation of human dendritic cells: a novel mechanism of immune evasion. J. Immunol. Baltim. Md 1950.

[48] Pascutti MF (2011). Interplay between modified vaccinia virus Ankara and dendritic cells: phenotypic and functional maturation of bystander dendritic cells. J. Virol..

[49] Brüning A, Runnebaum IB (2003). CAR is a cell-cell adhesion protein in human cancer cells and is expressionally modulated by dexamethasone, TNFalpha, and TGFbeta. Gene Ther..

[50] Batich KA (2017). Long-term Survival in Glioblastoma with Cytomegalovirus pp65-Targeted Vaccination. Clin. Cancer Res. Off. J. Am. Assoc. Cancer Res..

[51] Prins RM, Cloughesy TF, Liau LM (2008). Cytomegalovirus immunity after vaccination with autologous glioblastoma lysate. N. Engl. J. Med..

[52] Mitchell DA (2008). Sensitive detection of human cytomegalovirus in tumors and peripheral blood of patients diagnosed with glioblastoma. Neuro-Oncol..

[53] Nauerth M (2013). TCR-ligand koff rate correlates with the protective capacity of antigen-specific CD8+ T cells for adoptive transfer. Sci. Transl. Med..

[54] Wadell G, de Jong JC (1980). Restriction endonucleases in identification of a genome type of adenovirus 19 associated with keratoconjunctivitis. Infect. Immun..

[55] Perdiguero B (2015). Virological and immunological characterization of novel NYVAC-based HIV/AIDS vaccine candidates expressing clade C trimeric solublegp140(ZM96) and Gag(ZM96)-Pol-Nef(CN54) as virus-like particles. J. Virol..

[56] Ruzsics Z, Lemnitzer F, Thirion C (2014). Engineering adenovirus genome by bacterial artificial chromosome (BAC) technology. *Methods Mol*. Biol. Clifton NJ.

