# Supplementary material for: Vaccine vectors based on Adenovirus 19a/64 exhibit broad cellular tropism and potently restimulate HCMV-specific T cell responses ex vivo
Source: Sci Rep. 2018 Jan 24;8:1474. doi: 10.1038/s41598-018-19874-1 (PMC5784015; doi:10.1038/s41598-018-19874-1)
Supplement: Supplementary file 1 — Supplementary Information [file 41598_2018_19874_MOESM1_ESM.pdf]

Supplementary information

Vaccine vectors based on Adenovirus 19a/64 exhibit broad cellular tropism and potently restimulate HCMV-specific T cell responses ex vivo

Richard Kiener[1], Markus Fleischmann[1], Christiane Schwegler[1], Zsolt Ruzsics[3], Christian Thirion[2], Silke Schrödel[2], Benedikt Asbach[1], Ralf Wagner[1,4*]

[1] Institute of Medical Microbiology and Hygiene, Universität Regensburg, Franz-Josef-Strauß-Allee 11, 93053 Regensburg, Germany

[2] SIRION Biotech GmbH, Am Klopferspitz 19, 82152, Martinsried, Germany

[3] Institute of Virology, Medical Center – University of Freiburg, Medical Faculty, University of Freiburg, Hermann-Herder Str 11, 79104 Freiburg, Germany

[4] Institute of Clinical Microbiology and Hygiene, University Hospital Regensburg, Franz-Josef- Strauß-Allee 11, 93053 Regensburg, Germany

[*] corresponding author: Prof. Dr. Ralf Wagner, Molecular Microbiology (Virology), Institute of Medical Microbiology and Hygiene, Franz-Josef-Strauss-Allee 11, University of Regensburg, 93053 Regensburg

phone: +49 941 944 6452, fax: +49 941 944 6484

email: ralf.wagner@klinik.uni-regensburg.de

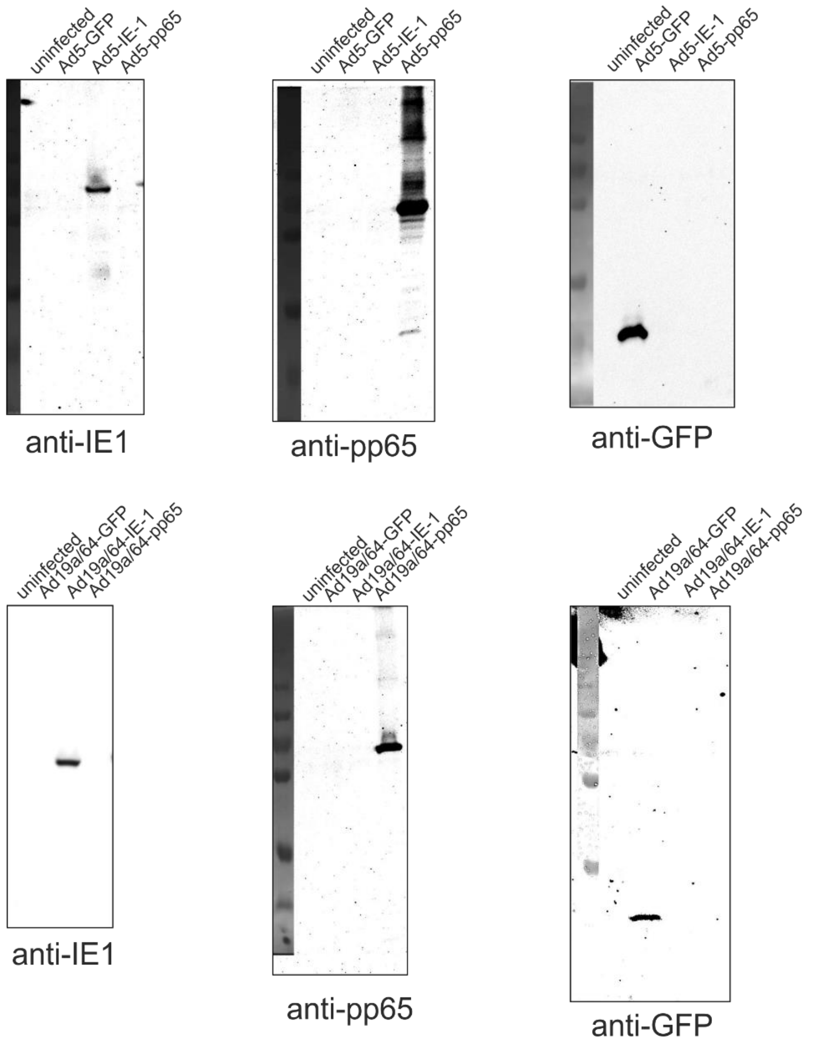

Supplementary Figure 1 Western Blot analysis of transgene expression 48 hours post infection (hpi). Cropped parts of these blots are displayed in Figure 1A. HEK293T cells were infected at a multiplicity of infection (MOI) of 10 with Ad5 or Ad19a/64 vectors expressing the genes IE-1, pp65, or GFP as indicated.

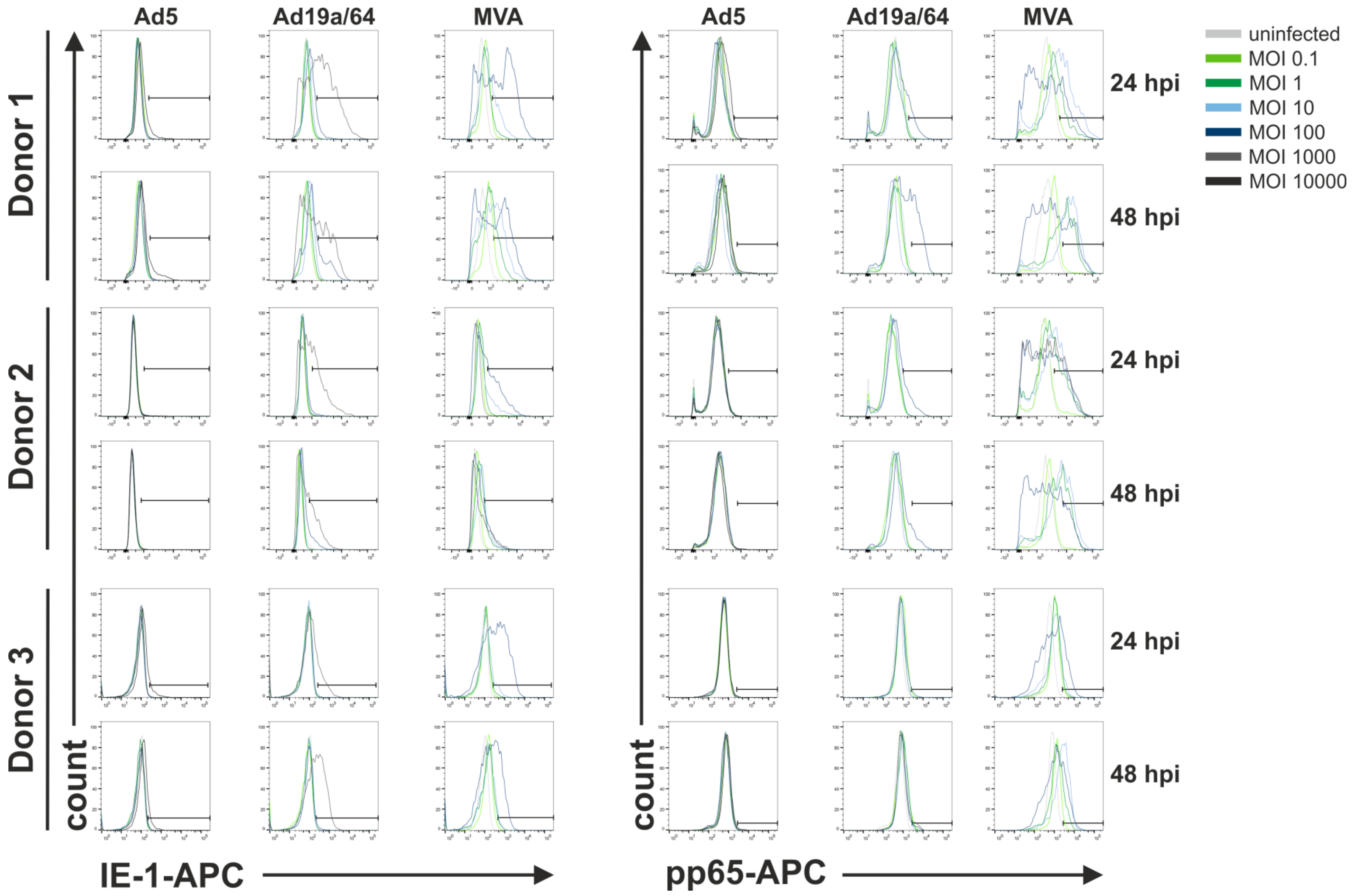

Supplementary Figure 2 MoDCs from 3 different HCMV seronegative blood donors were infected at the indicated MOIs with IE-1- or pp65-expressing vectors and transgene expression was analyzed at the indicated time points via flow cytometry after intracellular antigen staining. The gating was performed as shown in figure 3.
